# Survey dataset on socioeconomic status and artificial reef fishing activity on Terengganu coastal water

**DOI:** 10.1016/j.dib.2024.110028

**Published:** 2024-01-05

**Authors:** Nur Azura Sanusi, Normi Azura Ghazali, Roseliza Mat Alipiah, Roshanim Koris, Razak Zakariya

**Affiliations:** a Higher Institution Center of Excellence (HICoE), Institute of Tropical Aquaculture and Fisheries, Universiti Malaysia Terengganu, 21030 Kuala Nerus, Terengganu, Malaysia; b Faculty of Business, Economics and Social Development, Universiti Malaysia Terengganu, 21030 Kuala Nerus, Terengganu, Malaysia; c Faculty of Science and Marine Environment, Universiti Malaysia Terengganu, 21030 Kuala Nerus, Terengganu, Malaysia

**Keywords:** Fishing capital, Socioeconomic benefits, Artisanal fishers, Artificial habitat

## Abstract

An artificial reef (AR) programme is being undertaken by the local fisheries authority in Terengganu, Malaysia, in an effort to mitigate the depletion of fish stocks in the coastal zone. This program is intended to protect inshore fishery habitats from trawls to increase fishery resources and improve the economic conditions of artisanal fishing communities. This article aims to present data on fishers’ demographic characteristics and artificial reef fishing activity on Terengganu coastal water. Primary data were collected using stratified sampling that involved 430 respondents from four fishing communities in Terengganu, namely Setiu, Marang, Dungun and Kemaman. The dataset was obtained through a self-structured questionnaire. Data analysis and summary are presented using tables and figures. The findings provide valuable feedback on the socio-economic impact and economic value of artificial reefs to the fishermen and can be useful for policymakers to prevent the over-exploitation of fishery resources in Malaysian marine territories.

Specifications Table

**Table:** 

Subject	Economics
Specific subject area	Fisheries Economics
Data format	Raw, Analyzed, Descriptive, Statistical
Type of data	Table, Chart, Graph, Figure, Text
Data collection	Data were obtained through a structured questionnaire (face-to-face interview) for the target group, which is the fishing communities in districts of Setiu, Marang, Dungun and Kemaman in Terengganu coastal waters. The data were collected from August to October 2022, using disproportionate stratified random sampling. The enumerators asked and filled out the questionnaire on site. A total of 430 responses were obtained by the survey.
Data source location	Fishing activities at artificial reef areas in coastal waters of Setiu, Marang, Dungun and Kemaman in Terengganu.
Data accessibility	**R**epository name: Mendeley Data Data identification number: 10.17632/jygfjf449d.1 Direct URL to data: https://data.mendeley.com/datasets/jygfjf449d/1[5]

## 1 Value of the Data

• The dataset provides valuable insights into the socioeconomic status of the local communities in Terengganu. It can provide a deeper understanding of various factors, including income levels, education, employment, and access to resources. Researchers can analyse how these socioeconomic factors correlate with artificial reef fishing activity, providing a comprehensive understanding of the human dimension of coastal communities.
• The dataset has broad applicability, and its benefits extend to those involved in policymaking, environmental conservation, research, education, community development, and various sectors that intersect with the complex dynamics of socioeconomic and fishing activities in Terengganu coastal waters.
• Researchers from different disciplines, such as marine biology, sociology, economics, and environmental science, can collaborate and combine the dataset with their own to conduct cross-disciplinary studies. This approach can provide a more comprehensive understanding of the complex interactions between human activities and coastal ecosystems.
• In addition to the socioeconomic characteristics of fishermen, there is a lack of research on artificial reefs in Malaysia. Ecological study, particularly pertaining to fishing activities and artificial reefs, is infrequent. Due to the infrequency of such examinations, conducting a thorough assessment is crucial for identifying any deficiencies. These data pieces offer comprehensive insights into the importance, requirements, and roles of artificial reefs. The assessment of the crucial role played by artificial reef fishing activity is infrequently conducted.

## 2 Data Description

**Fig. 1:**
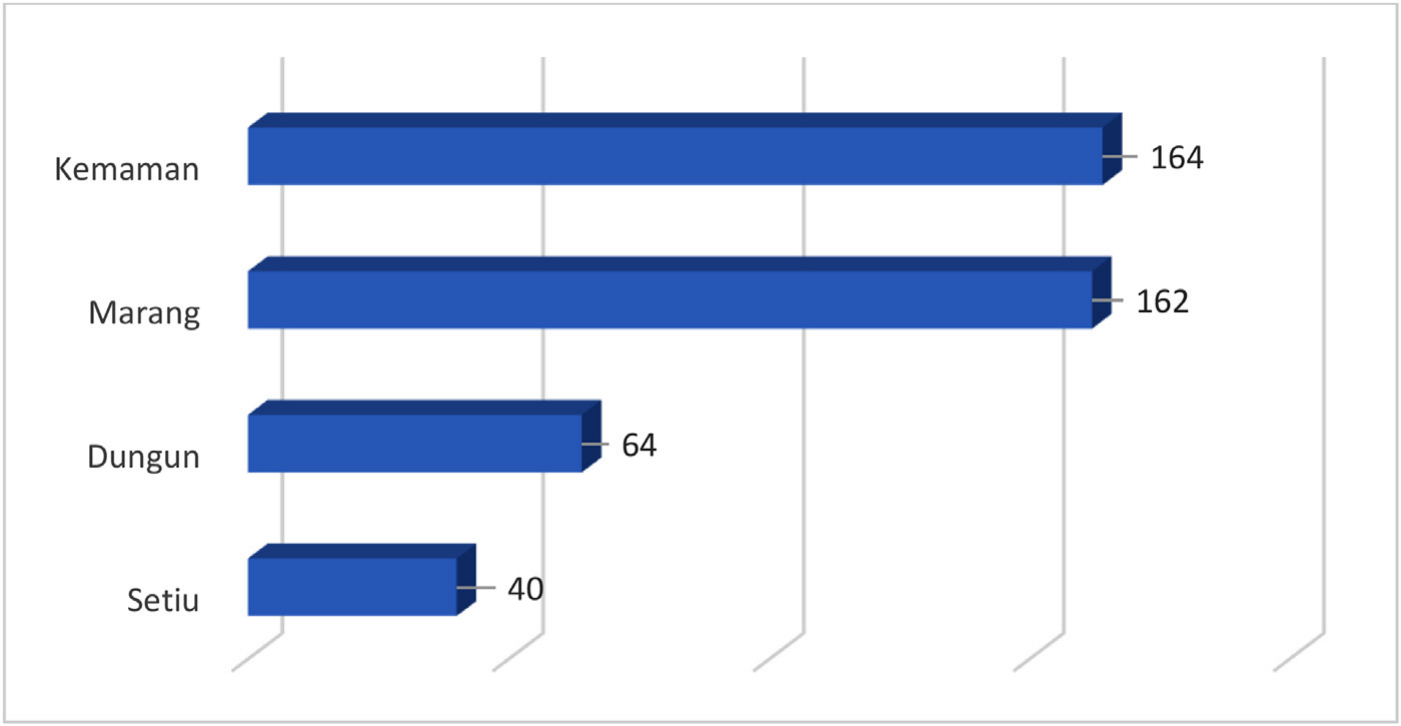
Number of respondents according to selected fishing district in Terengganu.

Within the data repository Mendeley Dataset [5], the researcher provides two files that consists of survey questionnaire in pdf format and Microsoft Excel file format, include the raw data of the survey and the related codebook pertaining to the variables encompassing the survey that conducted on socioeconomic status and artificial reef fishing activities in the coastal waters of Terengganu. The initial data was provided in the Microsoft Excel file format [5]. Additionally, the researcher shares the English versions of the questionnaire files that have been used in the survey [5]. Terengganu is located on the east coast of Peninsular Malaysia, and its coastal waters extend along the South China Sea. The precise coordinates of Terengganu's coastal waters can vary, but generally, it stretches from approximately 4.0° north latitude to 5.5° north latitude and from about 102.0° east longitude to 104.5° east longitude. From 2018 to 2022, a total of 150 artificial reefs have been deployed in Terengganu coastal waters [1]. This survey was conducted on selected fishing communities in the coastal waters area of Setiu, Marang, Dungun and Kemaman districts in Terengganu. The inclusion of only these four districts in the survey was based on the data of artificial reefs area provided by the Fisheries Development Authorities of Malaysia (FDAM) [2]. This data indicated that both fisheries artificial reefs and lobster artificial reefs are deployed in the coastal waters of these districts. The objective of the survey was to examine the socioeconomic benefits of the artisanal fishers' programme for artificial reefs in Terengganu State, which has the most number of artificial structures in Peninsular Malaysia. A total of 430 fishermen were interviewed and responded to questionnaires. The highest number of respondents are from Kemaman district, which 164 fishermen and were followed by Marang district (see Fig. 1).

There are many types of artificial reefs that been deployed in Terengganu coastal waters by the Department of Fisheries Malaysia (DOFM) and the Fisheries Development Authorities of Malaysia (FDAM), and also the state government. Meanwhile, the different types of artificial reefs have been designed to accommodate different kinds of fish. Different artificial reef models served as alternative habitats for feeding, sheltering, and protecting the marine species [8]. Table 1 shows the numbers and types of artificial reefs that been found by the respondents in their fishing areas.

**Table 1:** Types of artificial reef structures in Terengganu coastal waters by fishing districts.

Type of artificial reefs	Setiu		Dungun		Marang		Kemaman		Total	
	Frequency	Percent (%)	Frequency	Percent (%)	Frequency	Percent (%)	Frequency	Percent (%)	Frequency	Percent (%)
Ceramic reef	–	–	–	–	7	4.3	1	0.6	8	1.9
Concrete reef	5	12.5	1	1.6	1	0.6	5	3.0	12	2.8
Cube reef	–	–	12	18.8	49	30.2	31	18.9	92	21.4
Cuboid reef	14	35.0	16	25.0	13	8.0	28	17.1	71	16.5
Cylinder reef	8	20.0	2	3.1	2	1.2	2	1.2	14	3.3
Juvenile soft based reef	–	–	18	28.1	46	28.4	19	11.6	83	19.3
Lobster reef	1	2.5	2	3.1	–	–	2	1.2	5	1.2
Oil rig reef	–	–	–	–	10	6.2	–	–	10	2.3
Others	3	7.5	–	–	11	6.8	8	4.9	22	5.1
Recreation reef	–	–	8	12.5	3	1.9	1	0.6	12	2.8
Sunken ship reef	–	–	–	–	8	4.9	5	3.0	13	3
Tetrapod reef	–	–	1	1.6	9	5.6	7	4.3	17	4
Tire reef	9	22.5	4	6.3	3	1.9	55	33.5	71	16.5
Total	40	100.0	64	100.0	162	100.0	164	100.0	430	100

**Fig. 2:**
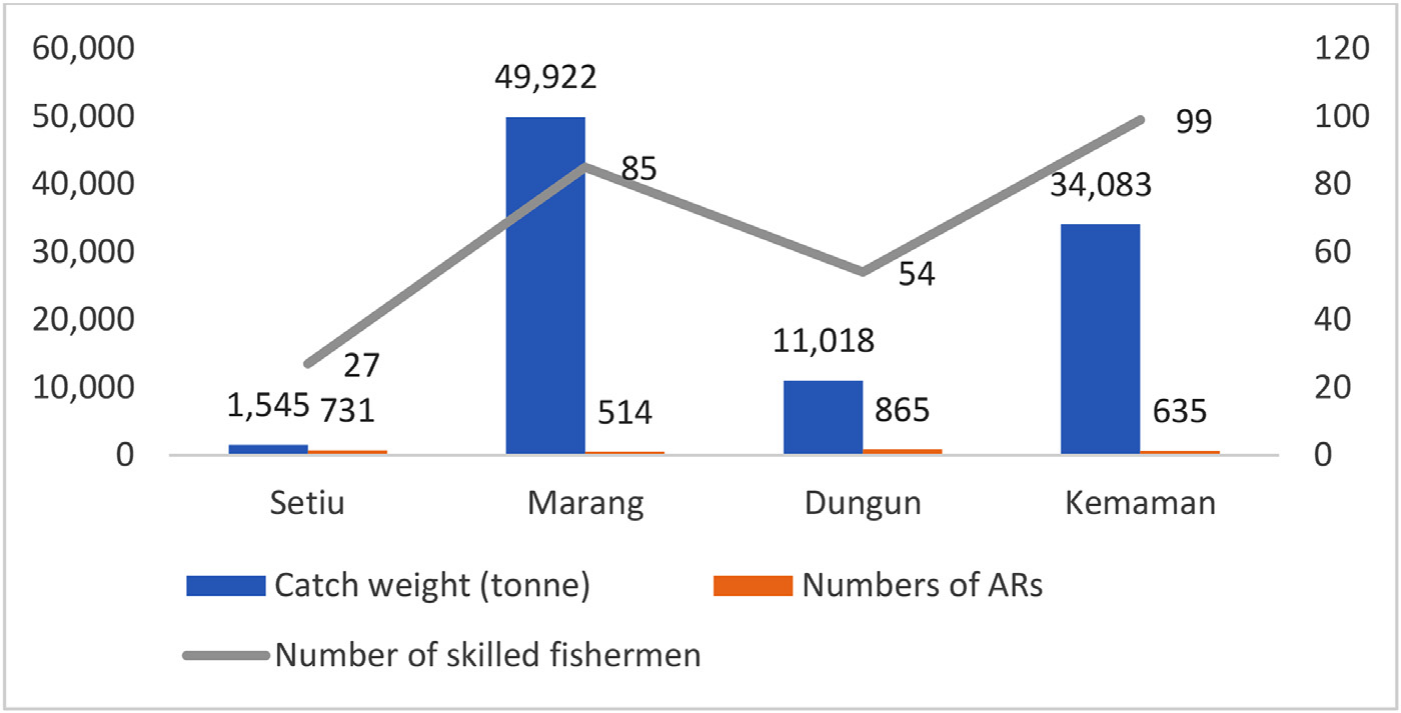
Total catches, numbers of AR and numbers of skilled fishermen by the fishing district.

Meanwhile, Fig. 2 shows the total catches, numbers of AR and numbers of skilled fishermen by the fishing district. Based on the analysis of frequency data at the district level, it was seen that Marang exhibited the largest catch (49, 922 tonnes) in comparison to other districts. Conversely, Setiu registered the lowest catch (1545 tonnes) despite having a greater number of artificial reefs anchored in its coastal waters compared to other districts. The observed discrepancy in total catches could potentially be attributed to the relatively small size of respondents involved in this study compared to other districts (see Fig. 1). However, according to [7], artificial reef areas were increasing the number of catches to the fishermen. On the other hand, further research is necessary to investigate various artificial reef models in order to protect and secure the long-term sustainability of marine species populations in proximity to artificial reefs [8]. Therefore, all types of artificial reefs are potential to increase the numbers of catch yields by the fishers.

**Fig. 3:**
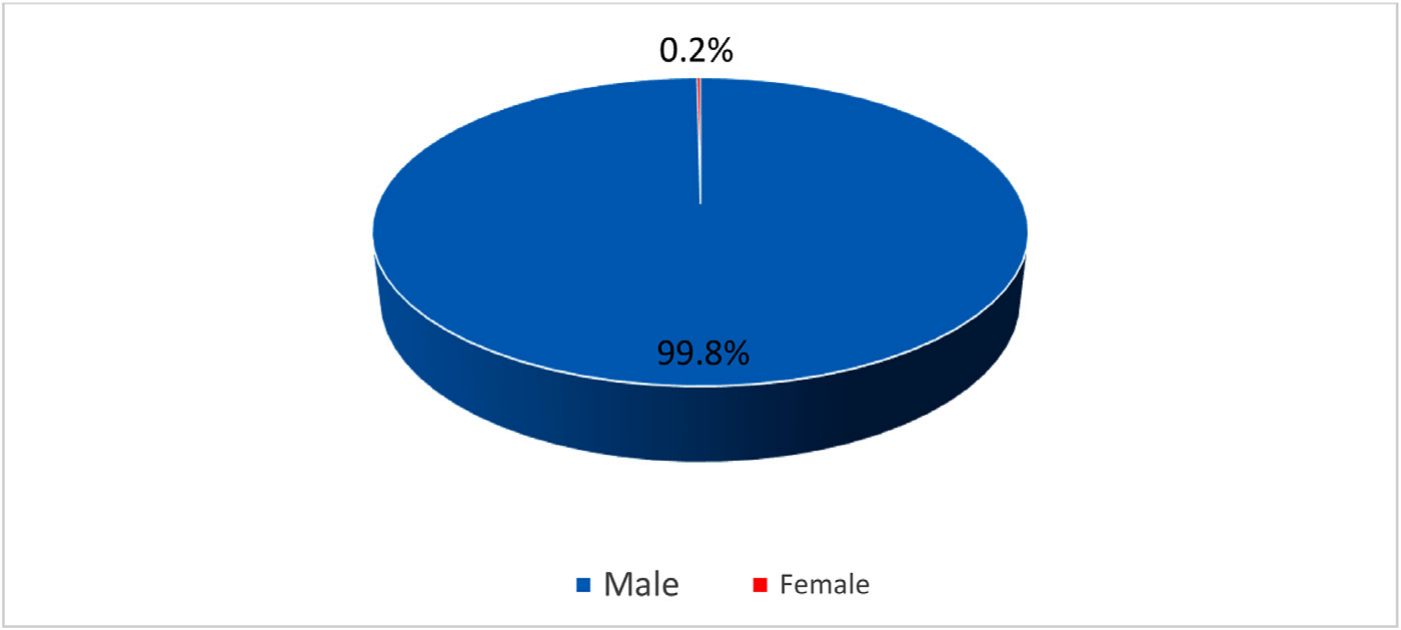
Gender of respondents.

**Fig. 4:**
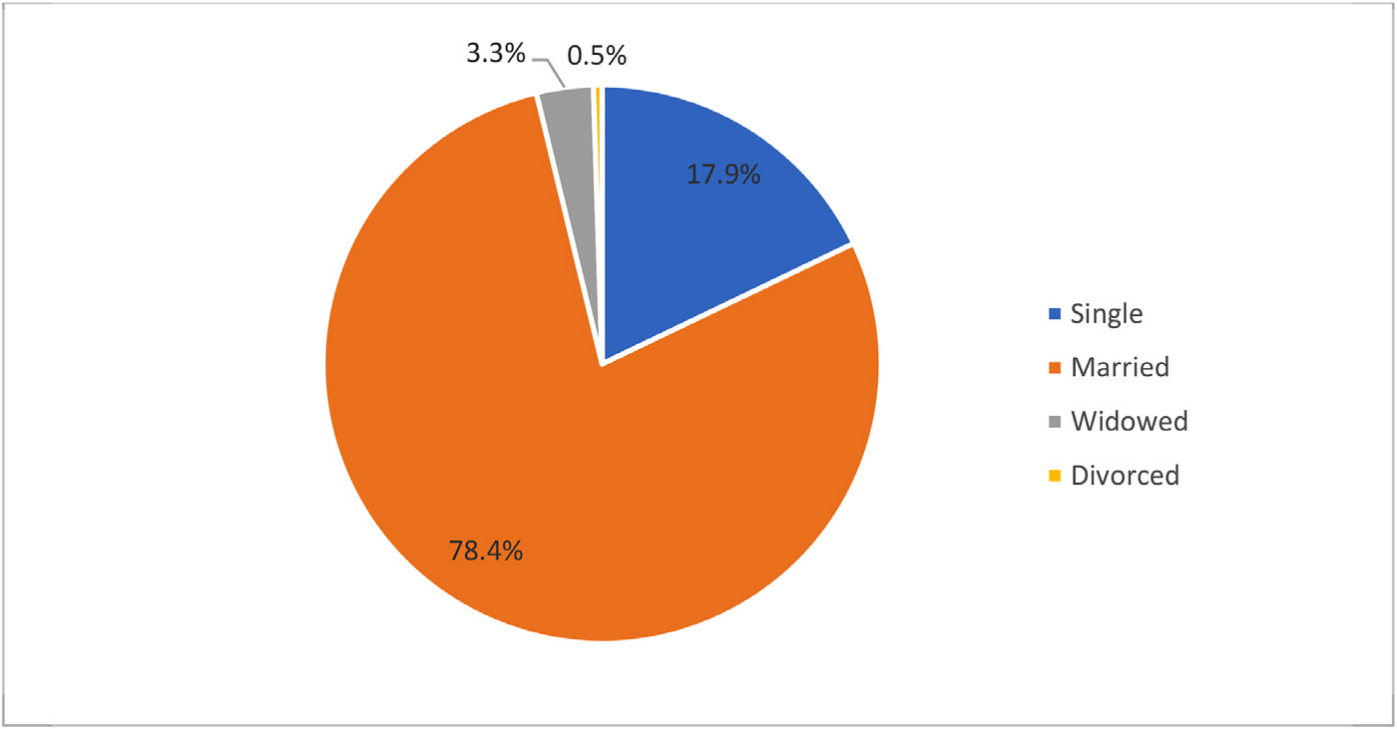
Marital status of respondents.

**Fig. 5:**
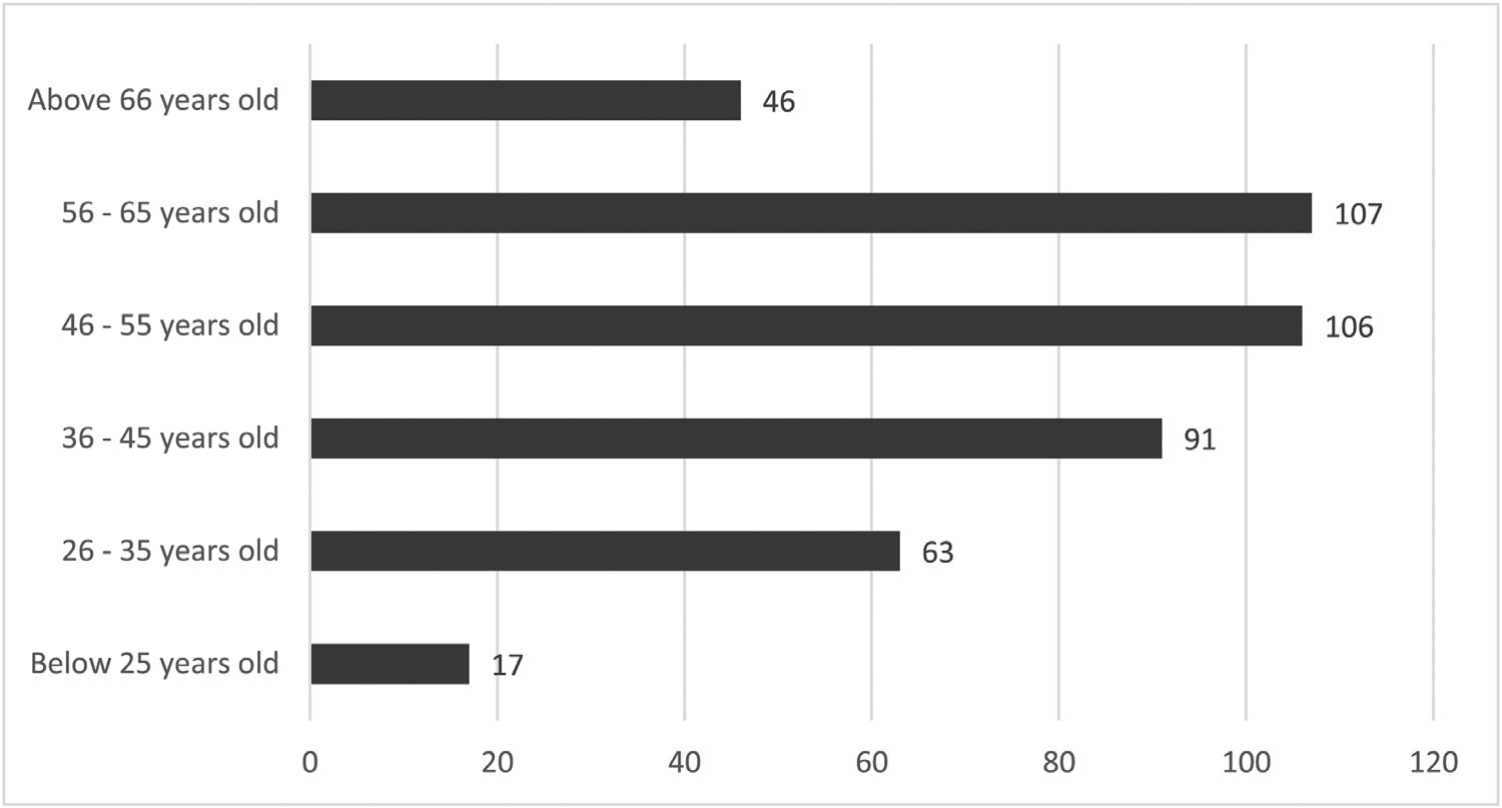
Age of respondents.

**Fig. 6:**
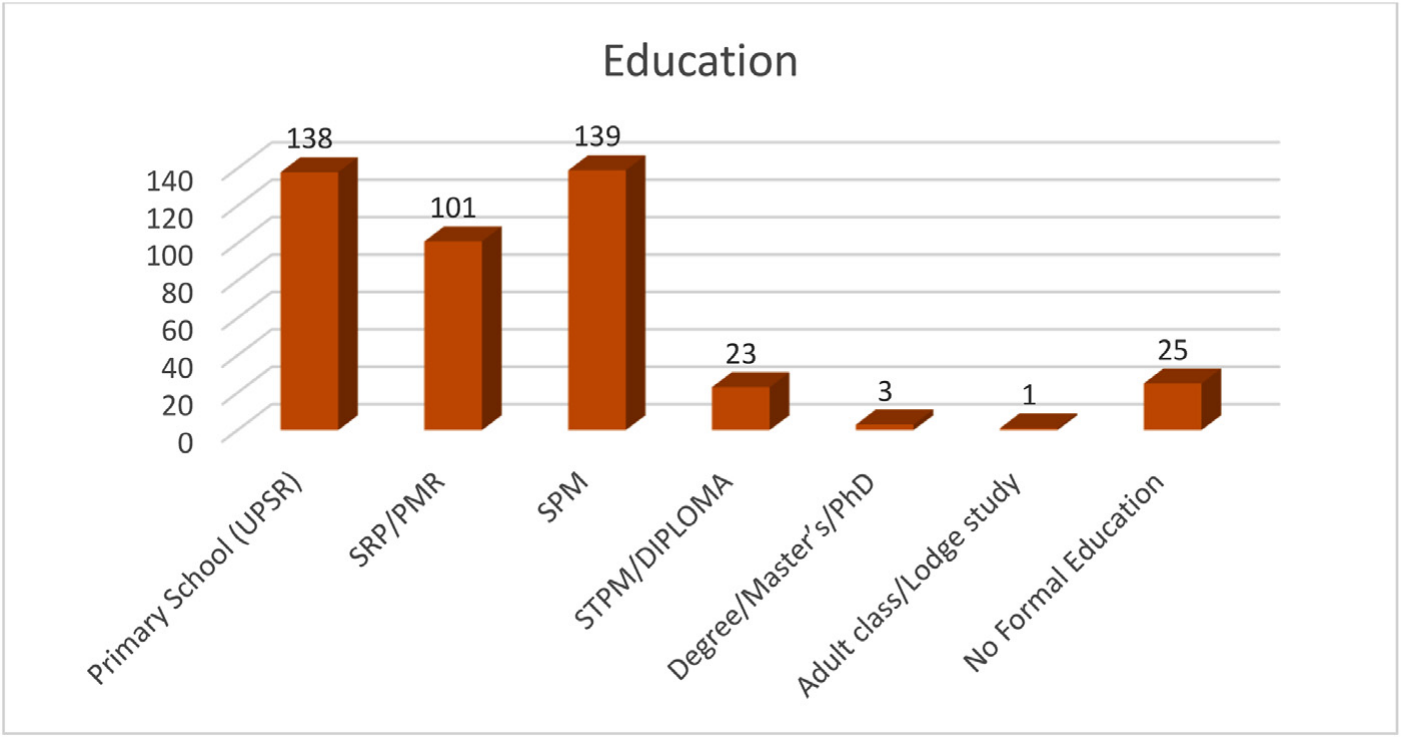
Educational level of respondents.

The statistics of the fishers’ demographic characteristics (i.e., age, gender, marital status and educational level) are shown in Figs. 3–6. Most respondents were married (see Fig. 4). Most respondents that involved with this study were over 35 years old (81.5%) (see Fig. 5). This matches national statistics, which indicates that the average age of Malaysian fishers is increasing, which the number of fishermen age 65 years and above has increased by more than 100% in ten years [3]. The survey also shows that most respondents received primary and secondary education (87.9%); only 5.8% had not received formal education. Meanwhile, 6.0% of respondents received higher-level education, STPM/Diploma and Degree/Master/PhD, which means that fisher households mostly have low education levels (see Fig. 6).

The research study revealed that a majority of the fishers were skilful and experience in fishing activities (61.6%) (see Table 2). Therefore, this proves the previous assumption that fishing is the most important income source for artisanal fishers in the study area. Furthermore, fishers’ experience in fishing activities is important to understand the households’ income and dependency on fishing activities for their livelihoods [4]. The experience of fisherman is depending on their early involvement in the field and the level of education they possess. Fishermen aged 50 and above have typically received only primary education [7].

**Table 2:** Distributions of fishing capital.

Items	Frequency (*N* = 430)	Percentage
1. Types of fishermen Boat owner Artisanal fisher Skipper Boat crews Others	240 3 59 126 2	55.8% 0.7% 13.7% 29.3% 0.5%
2. Fishing skilled Very skillful Skillful Moderate Less skillful	185 80 126 39	43.0% 18.6% 29.3% 9.1%
3. Fishing zone Zone A Zone B Zone C Zone A and B Zone A, B and C Not disclose the zone	340 57 7 9 1 16	79.1% 13.3% 1.6% 2.1% 0.2% 3.7%
4. Equipment used to locate AR areas GPS Buoy Echo sounders Traditional	268 10 52 100	62.3% 2.3% 12.1% 23.3%

Table 3 shows that 374 fishers were using outboard-powered vessels and 56 fishers were using inboard-powered vessels. Majority of traditional fishermen rely on outboard engines. This is due to the utilization of outboard engines that are appropriate for the open fiberglass boat [7]. On average, fishers who use inboard-powered vessels (17.8 miles) travel farther than outboard fishers (10.1 miles) to their fishing venues. This is because fishers with outboard-powered vessels are restricted to fishing near the shoreline, while fishers with inboard-powered vessels can go much farther out to sea.

**Table 3:** Distance from jetty to fishing area and artificial reef.

Vessel Type	Mean (Miles nautical)	Standard Deviation	Frequency
Outboard	10.1	9.9	374
Inboard	17.8	19.2	56
Total	11.2	12.0	430

Table 4 shows the different fishing operations and total catches during the main season by vessel type in AR areas. Generally, fishing operations with inboard-powered vessels generate more yield than those with outboard-powered vessels. The study findings by [9] indicated that fishers using boats with inboard-powered vessels were able to generate a substantial income from their catch. However, [9] also reveal that the fibreglass outboard-boats (sampan) which commonly used by artisanal fishers are currently equipped with powerful engines (15 horsepower and above) to facilitate extensive fishing operations. The mean crew size for inboard-powered vessels is 9.1, which is much higher than the mean crew size for outboard-powered vessels of just 2.3. Similar to crew size, the means of total catch (687.3 kg) and total sales (MYR 23,789.60) are higher for inboard-powered vessels compared to the mean total catch (155.3 kg) and total sales (MYR 11,142.60) of outboard-powered vessels. In addition, the mean total fishing trips per month (19.7 times) of inboard-powered vessels is also higher than those of outboard-powered vessels (13.9 times).

**Table 4:** Fishing operations and catch value during the main season by vessel type in AR areas.

Vessel Type	Statistic	Crew Size	Fishing Trip/ month	Catch (kg/month)	Sales (MYR/month)
Outboard	Mean	2.3	13.9	155.3	11,142.6
	Standard Deviation	3.7	9.8	320.5	24,407.8
	Frequency*(*N* = 374)	374	374	374	374
Inboard	Mean	9.1	19.7	687.3	23,789.6
	Standard Deviation	8.0	6.5	1726.0	58,649.1
	Frequency*(*N* = 56)	56	56	56	56

⁎ Total respondents, *N* = 374 + 56 = 430 fishermen.

The researcher found that most fishers are very skilful (185 fishers) or skilful (80 fishers) in catching fishes. Table 5 shows that skilful fishers have the highest catch (278.3 kg), followed by those with moderate skilful fishers (245.8 kg), very skilful (198.5 kg) and less skilful (169.8 kg). However, highly skilful and experienced fishers have high sales (MYR 17,290.40) but lower income (MYR 1487.70) per month. Fishers with skilful, moderate and less skilful have lower sales than very skilful fishers (MYR 10,818.10, MYR 8809.70 and MYR 8342.70, respectively). Moderate skilful fishers have high income (MYR 1887.40), followed by skilful fishers (MYR 1540.60) and less skilful fishers (MYR 1329.50) per month.

**Table 5:** Fishing skills with total catch and sales in AR areas.

Fishing skill	Statistic	Catch (kg/month)	Sales (MYR/month)	Income (MYR/month)
Less skillful	Mean	169.8	8342.7	1329.5
	Standard Deviation	207.6	14,633.6	1269.4
	Frequency *(*N* = 39)	39	39	39
Moderate	Mean	245.8	8809.7	1887.4
	Standard Deviation	605.9	20,367.8	2720.5
	Frequency *(*N* = 126)	126	126	126
Skillful	Mean	278.3	10,818.1	1540.6
	Standard Deviation	856.7	20,321.5	1138.0
	Frequency *(*N* = 80)	80	80	80
Very skillful	Mean	198.5	17,290.4	1487.7
	Standard Deviation	772.9	41,686.2	1406.6
	Frequency *(*N* = 185)	185	185	185

⁎ Total respondent, *N* = 39 + 126 + 80 + 185 = 430 fishermen.

Table 6 shows the different fishing gears by vessel type used by the fishers in AR areas. Fishers with inboard-powered vessels use various fishing methods and techniques, such as trawl nets, fish purse seines, drift/gill nets and other seines. Meanwhile, fishers with outboard-powered vessels used drift/gill nets, hooks and lines, traps and other seines. Fishers with inboard-powered vessels had higher catches using trawl nets (1835 kg) and fish purse seines (36,342 kg). Compared to inboard-powered vessel fishers, outboard-powered vessel fishers recorded higher catches using drift/gill nets (42,894 kg) and hooks and lines (7596 kg).

**Table 6:** Fishing gear by vessel type in AR areas.

Fishing gear	Outboard		Inboard	
	Frequency (total fishers’)	Total Catch/ month (kg)	Frequency (total fishers’)	Total Catch/ month (kg)
Trawl nets	–	–	16	1835
Fish Purse Seines	–	–	36	36,342
Drift/Gill Net	226	42,894	2	120
Hooks & Lines	75	7596	–	–
Other Seines	37	1691	2	193
Traps	36	5897	–	–
Total (*N* = 430)	374		56	

During the early phases of the research and construction of artificial reefs in Malaysia in 1975, the financial costs were quite low, and the exact number was not explicitly mentioned [10]. Between 2006 and 2010, the Federal Government has earmarked up to MYR28 million for the establishment of 115 artificial reef sites across the nation's waterways [11]. The Department of Fisheries has been allocated a budget of MYR6.5 million by the central government to facilitate the effective development and oversight of artificial reefs in the waters of Peninsular and Sabah. This fund will be allocated and used throughout the timeframe spanning from 2021 to 2023. For the first six months in 2023, the Department of Fisheries has allocated MYR1.5 million to implement the deployment of 57 units of trawler barrier juvenile reef for the purpose of reef protection. The focus of these endeavors will be directed towards five specific states, specifically Terengganu, Kelantan, Johor, Kedah, and Selangor [12]. The artificial reefs program led to a 10 percent increase in the total catch of coastal fishermen in Terengganu from January to April 2022 [13].

## 3 Experimental Design, Materials and Methods

This research involved face-to-face surveys with 430 respondents from fishing communities in Setiu, Dungun, Marang, and Kemaman districts in Terengganu. The researcher used disproportionate stratified sampling as a tool for the respondent selection. The respondents for this study were selected based on the percentage of catch landings in each district, as shown in Fig. 1. Stratified sampling divides the population into homogeneous, mutually exclusive subgroups known as strata [6]. With reference to Fig. 2, the strata in concern classify different AR areas, namely Setiu, Marang, Dungun, and Kemaman. The method of sampling involves specifically selecting individuals who have engaged in fishing activities within the designated artificial reefs area. In the event that the fisherman has not previously catches within the designated artificial reefs area, the fisherman shall be excluded as a participant in the study. The designated enumerators will conduct in-person interviews with fishermen at fishing jetties. Before asking the survey questions, the enumerator will initially provide an explanation to the respondents regarding the purpose of the study. Data were collected using a structured questionnaire designed by the researcher [5].

The questionnaire has three main sections and five sub-sections. Section A was about the fishery activities information, such as information on artificial reef (Section A1); information on fishing effort at the artificial reef area (Section A2) and catch results information (Section A3). Meanwhile, Section B was about vessel information and operation costs, vessel information, features and types of equipment/fishing gear (B1) and technology and the safety equipment owned by respondents in the vessels (B2). Lastly, Section C was about the respondents’ demographics such as age, gender, marital status, education level, income, role as fishermen and experience as fishermen. The raw data from the survey were quantitatively analyzed using the Statistical Package for the Social Sciences (SPSS) version 21 and Microsoft Excell. The data are presented in figures and tables.

## Limitations

None.

## Ethics statement

Here, we assure that the data collection was based on the respondents’ willingness to answer the questionnaire. All respondents involved in this survey were fully informed and they expressed consent with anonymous data collection before questioning.

## CRediT authorship contribution statement

**Nur Azura Sanusi:** Conceptualization, Methodology, Writing – original draft, Writing – review & editing, Supervision, Funding acquisition. **Normi Azura Ghazali:** Visualization, Formal analysis, Writing – review & editing. **Roseliza Mat Alipiah:** Writing – review & editing. **Roshanim Koris:** Writing – review & editing. **Razak Zakariya:** Writing – review & editing.

## Data Availability

Socioeconomic status and economic impact of artificial reefs (Original data) (Mendeley Data)
